# Crystal structure of di­chlorido­bis­(1,3,4,5-tetra­methyl-1*H*-imidazol-2-ium-2-thiol­ate-κ*S*)nickel(II)

**DOI:** 10.1107/S2056989015012281

**Published:** 2015-07-04

**Authors:** Aziza Ahmida, Ulrich Flörke, Hans Egold, Gerald Henkel

**Affiliations:** a Department Chemie, Fakultät für Naturwissenschaften, Universität Paderborn, Warburgerstrasse 100, D-33098 Paderborn, Germany

**Keywords:** crystal structure, nickel(II) complex, 1,3,4,5-tetra­methyl­imidazole-2-thione ligand

## Abstract

In the mol­ecular structure of the title compound, [NiCl_2_(C_7_H_12_N_2_S)_2_], the Ni^II^ atom has a distorted tetra­hedral geometry, coordinated by two Cl atoms [Ni—Cl= 2.2336 (6) Å] and two thione S atoms [Ni—S= 2.3024 (6) Å]. The angles at the Ni^II^ cation, which lies on a twofold rotation axis, are Cl—Ni—Cl = 115.58 (3)° and S—Ni—S = 94.55 (3)°. All other angles at the central Ni^II^ atom range from 109.46 (2) to 112.96 (2)°. The C—S—Ni angle is 99.91 (6)°. The planes of two imidazolium rings make a dihedral angle of 70.56 (6)°.

## Related literature   

For structures of related Ni complexes, see: Flörke *et al.* (2014 ▸); O’Neill *et al.* (1981 ▸). For the ability of *N*,*N*-di­methyl­imidazole­thione derivatives to act as effective anti-oxidants, see: Bhabak & Mugesh (2010 ▸); Yamashita & Yamashita (2010 ▸). For C—S bond lengths, see: Williams *et al.* (1997 ▸).
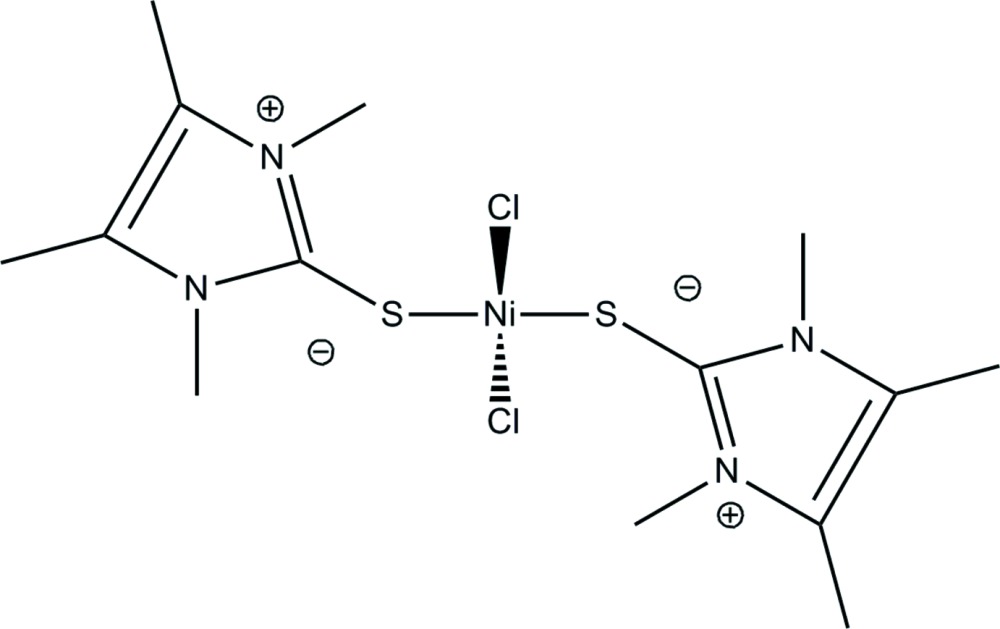


## Experimental   

### Crystal data  

[NiCl_2_(C_7_H_12_N_2_S)_2_]
*M* *_r_* = 442.10
Monoclinic, 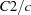

*a* = 14.8539 (17) Å
*b* = 8.5969 (10) Å
*c* = 16.4434 (19) Å
β = 112.104 (2)°
*V* = 1945.5 (4) Å^3^
*Z* = 4
Mo *K*α radiation
μ = 1.49 mm^−1^
*T* = 120 K
0.43 × 0.20 × 0.14 mm

### Data collection  

Bruker SMART CCD area-detector diffractometer
Absorption correction: multi-scan (*SADABS*; Sheldrick, 2004 ▸) *T*_min_ = 0.567, *T*_max_ = 0.819
8874 measured reflections
2396 independent reflections
2050 reflections with *I* > 2σ(*I*)
*R*_int_ = 0.033

### Refinement  

*R*[*F* ^2^ > 2σ(*F* ^2^)] = 0.035
*wR*(*F* ^2^) = 0.089
*S* = 1.09
2396 reflections
109 parameters
H-atom parameters constrained
Δρ_max_ = 0.56 e Å^−3^
Δρ_min_ = −0.27 e Å^−3^

### 

Data collection: *SMART* (Bruker, 2002 ▸); cell refinement: *SAINT* (Bruker, 2002 ▸); data reduction: *SAINT*; program(s) used to solve structure: *SHELXTL* (Sheldrick, 2008 ▸); program(s) used to refine structure: *SHELXTL*; molecular graphics: *SHELXTL*; software used to prepare material for publication: *SHELXTL* and local programs.

## Supplementary Material

Crystal structure: contains datablock(s) I, global. DOI: 10.1107/S2056989015012281/hp2071sup1.cif

Structure factors: contains datablock(s) I. DOI: 10.1107/S2056989015012281/hp2071Isup2.hkl

**Fig. 1.**
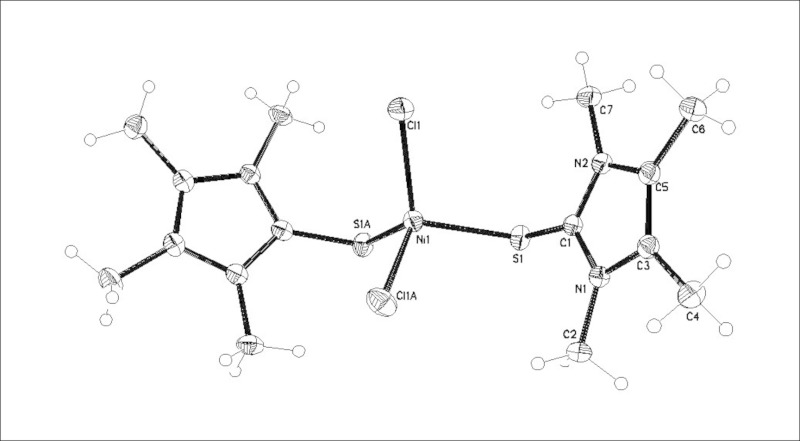
Molecular structure of the title compound with anisotropic displacement parameters drawn at the 50% probability level.

. DOI: 10.1107/S2056989015012281/hp2071fig1.tif
Mol­ecular structure of the title compound with anisotropic displacement parameters drawn at the 50% probability level.

CCDC reference: 1408996

Additional supporting information:  crystallographic information; 3D view; checkCIF report

## supplementary crystallographic information

### S1. Structural commentary

We are inter­ested in the chemistry of N,N-di­methyl­imidazole-thione derivatives due to their ability to act as effective anti­oxidants Bhabak *et al.* (2010); Yamashita *et al.* (2010). Complexes of the l-methyl derivative of imidazoline-2-thione with Zn(II) and Ni^II^ have been previously reported Flörke *et al.* (2014); O Neill *et al.*, (1981). In our group we are inter­ested in this type of compound to be used in the synthesis of biomimetic complexes. Here we report the synthesis of Ni^II^ chloride complex with 1,3,4,5-tetra-methyl­imidazole-2-thione ligands. The title compound shows the same trans configuration as the di­chloro­biis(1,3-diiso­propyl-methyl-1H-2H.imidazole-2-thione-S)zinc(II) (Flörke *et al.*, 2014). The Ni atom shows a distorted tetra­hedron with two chlorine atoms and two thione ligands, in which the angles at the Nickel cation are Cl—Ni—Cl 115.57 (3)° and S—Ni—S 94.55 (3)° . All other angles at the central Ni atom range from 109.46 (2)° to 112.96 (2)°. The Ni—S bond length is 2.2336 (6) Å. The C—S bond length in the title compound is elongated to 1.719 (2)Å by coordination to Nickel and closer to a single bond 1.81Å than a double bond 1.56Å (Williams *et al.*, 1997). The intra­molecular hydrogen bonds between the chlorine atom and hydrogen atoms of methyl group, H2a—-Cl and H7b—-Cl amount to 3.093 and 3.557.respectively.

### S2. Synthesis and crystallization

To a solution of 1,3,4,5-tetra-methyl­imidazoline-2-thione (0.390mg, 2.75mmol) in 40 ml aceto­nitrile, NiCl_2_ (0.162 mg, 1.25 mmol) ) was added and the mixture was stirred at room temperature for 24h. After removal of the solvent and subsequent drying in vacuum the residue was crystallized by diffusion of di­ethyl ether into a concentrated aceto­nitrile solution to give green single-crystals of the title complex.

### S3. Refinement

Crystal data, data collection and structure refinement details are summarized in Table 1. All H-atoms were clearly identified in difference syntheses and then refined at idealized positions riding on the carbon atoms with isotropic displacement parameters Uiso(H) = 1.5U(-CH_3_) and C–H 0.98 Å. All CH_3_ hydrogen atoms were allowed to rotate but not to tip.

### Crystal data

**Table:**

[NiCl_2_(C_7_H_12_N_2_S)_2_]	*F*(000) = 920
*M**_r_* = 442.10	*D*_x_ = 1.509 Mg m^−^^3^
Monoclinic, *C*2/*c*	Mo *K*α radiation, λ = 0.71073 Å
*a* = 14.8539 (17) Å	Cell parameters from 2710 reflections
*b* = 8.5969 (10) Å	θ = 2.7–28.3°
*c* = 16.4434 (19) Å	µ = 1.49 mm^−^^1^
β = 112.104 (2)°	*T* = 120 K
*V* = 1945.5 (4) Å^3^	Prism, green
*Z* = 4	0.43 × 0.20 × 0.14 mm

### Data collection

**Table:**

Bruker SMART CCD area-detector diffractometer	2396 independent reflections
Radiation source: sealed tube	2050 reflections with *I* > 2σ(*I*)
Graphite monochromator	*R*_int_ = 0.033
φ and ω scans	θ_max_ = 28.2°, θ_min_ = 2.8°
Absorption correction: multi-scan (*SADABS*; Sheldrick, 2004)	*h* = −19→19
*T*_min_ = 0.567, *T*_max_ = 0.819	*k* = −11→10
8874 measured reflections	*l* = −21→21

### Refinement

**Table:**

Refinement on *F*^2^	Primary atom site location: structure-invariant direct methods
Least-squares matrix: full	Secondary atom site location: difference Fourier map
*R*[*F*^2^ > 2σ(*F*^2^)] = 0.035	Hydrogen site location: difference Fourier map
*wR*(*F*^2^) = 0.089	H-atom parameters constrained
*S* = 1.09	*w* = 1/[σ^2^(*F*_o_^2^) + (0.0492*P*)^2^ + 0.3156*P*] where *P* = (*F*_o_^2^ + 2*F*_c_^2^)/3
2396 reflections	(Δ/σ)_max_ < 0.001
109 parameters	Δρ_max_ = 0.56 e Å^−^^3^
0 restraints	Δρ_min_ = −0.26 e Å^−^^3^

### Special details

**Table:**

Geometry. All e.s.d.'s (except the e.s.d. in the dihedral angle between two l.s. planes) are estimated using the full covariance matrix. The cell e.s.d.'s are taken into account individually in the estimation of e.s.d.'s in distances, angles and torsion angles; correlations between e.s.d.'s in cell parameters are only used when they are defined by crystal symmetry. An approximate (isotropic) treatment of cell e.s.d.'s is used for estimating e.s.d.'s involving l.s. planes.
Refinement. Refinement of *F*^2^ against ALL reflections. The weighted *R*-factor *wR* and goodness of fit *S* are based on *F*^2^, conventional *R*-factors *R* are based on *F*, with *F* set to zero for negative *F*^2^. The threshold expression of *F*^2^ > σ(*F*^2^) is used only for calculating *R*-factors(gt) *etc*. and is not relevant to the choice of reflections for refinement. *R*-factors based on *F*^2^ are statistically about twice as large as those based on *F*, and *R*- factors based on ALL data will be even larger.

### Fractional atomic coordinates and isotropic or equivalent isotropic displacement parameters (Å^2^)

**Table:**

	*x*	*y*	*z*	*U*_iso_*/*U*_eq_	
Ni1	0.0000	0.47837 (4)	0.2500	0.02273 (12)	
Cl1	0.05106 (4)	0.61687 (7)	0.16067 (3)	0.03670 (15)	
S1	0.11606 (3)	0.29666 (6)	0.32329 (3)	0.02693 (14)	
N1	0.22435 (11)	0.46213 (18)	0.46966 (10)	0.0225 (3)	
N2	0.27871 (11)	0.48030 (18)	0.36568 (10)	0.0235 (3)	
C1	0.20753 (13)	0.4178 (2)	0.38685 (12)	0.0222 (4)	
C2	0.16219 (14)	0.4241 (3)	0.51751 (13)	0.0292 (4)	
H2A	0.1144	0.5072	0.5092	0.044*	
H2B	0.2020	0.4135	0.5801	0.044*	
H2C	0.1283	0.3259	0.4954	0.044*	
C3	0.30709 (13)	0.5538 (2)	0.50124 (12)	0.0237 (4)	
C4	0.34399 (15)	0.6198 (2)	0.59158 (12)	0.0305 (4)	
H4A	0.3994	0.6881	0.5993	0.046*	
H4B	0.3646	0.5351	0.6345	0.046*	
H4C	0.2923	0.6798	0.6004	0.046*	
C5	0.34120 (13)	0.5658 (2)	0.43585 (12)	0.0250 (4)	
C6	0.42680 (14)	0.6509 (3)	0.43159 (14)	0.0338 (5)	
H6A	0.4624	0.7006	0.4883	0.051*	
H6B	0.4046	0.7306	0.3857	0.051*	
H6C	0.4697	0.5776	0.4180	0.051*	
C7	0.28996 (15)	0.4587 (3)	0.28167 (13)	0.0306 (5)	
H7A	0.3421	0.3837	0.2890	0.046*	
H7B	0.3063	0.5585	0.2619	0.046*	
H7C	0.2290	0.4196	0.2380	0.046*	

### Atomic displacement parameters (Å^2^)

**Table:**

	*U*^11^	*U*^22^	*U*^33^	*U*^12^	*U*^13^	*U*^23^
Ni1	0.02009 (19)	0.0228 (2)	0.02449 (19)	0.000	0.00747 (14)	0.000
Cl1	0.0332 (3)	0.0404 (3)	0.0408 (3)	0.0038 (2)	0.0189 (2)	0.0134 (2)
S1	0.0247 (2)	0.0242 (3)	0.0280 (3)	0.00122 (18)	0.00545 (19)	0.00048 (18)
N1	0.0203 (7)	0.0249 (9)	0.0229 (8)	0.0024 (6)	0.0087 (6)	0.0042 (6)
N2	0.0219 (8)	0.0263 (9)	0.0227 (8)	0.0042 (6)	0.0086 (6)	0.0054 (6)
C1	0.0209 (8)	0.0233 (10)	0.0228 (9)	0.0049 (7)	0.0085 (7)	0.0055 (7)
C2	0.0271 (10)	0.0386 (12)	0.0262 (10)	0.0002 (8)	0.0150 (8)	0.0044 (8)
C3	0.0219 (9)	0.0223 (10)	0.0260 (9)	0.0022 (7)	0.0078 (7)	0.0047 (7)
C4	0.0337 (11)	0.0298 (11)	0.0270 (10)	−0.0017 (8)	0.0101 (8)	0.0010 (8)
C5	0.0219 (9)	0.0253 (10)	0.0260 (9)	0.0015 (7)	0.0071 (7)	0.0057 (8)
C6	0.0275 (10)	0.0394 (12)	0.0354 (11)	−0.0042 (9)	0.0130 (8)	0.0091 (9)
C7	0.0314 (11)	0.0398 (12)	0.0247 (10)	0.0059 (9)	0.0153 (8)	0.0041 (9)

### Geometric parameters (Å, º)

**Table:**

Ni1—Cl1	2.2336 (6)	C2—H2C	0.9800
Ni1—Cl1^i^	2.2337 (6)	C3—C5	1.354 (3)
Ni1—S1^i^	2.3024 (6)	C3—C4	1.489 (3)
Ni1—S1	2.3024 (6)	C4—H4A	0.9800
S1—C1	1.719 (2)	C4—H4B	0.9800
N1—C1	1.344 (2)	C4—H4C	0.9800
N1—C3	1.386 (2)	C5—C6	1.492 (3)
N1—C2	1.458 (2)	C6—H6A	0.9800
N2—C1	1.343 (2)	C6—H6B	0.9800
N2—C5	1.388 (2)	C6—H6C	0.9800
N2—C7	1.464 (2)	C7—H7A	0.9800
C2—H2A	0.9800	C7—H7B	0.9800
C2—H2B	0.9800	C7—H7C	0.9800
			
Cl1—Ni1—Cl1^i^	115.58 (3)	C5—C3—C4	131.00 (18)
Cl1—Ni1—S1^i^	112.96 (2)	N1—C3—C4	122.16 (17)
Cl1^i^—Ni1—S1^i^	109.46 (2)	C3—C4—H4A	109.5
Cl1—Ni1—S1	109.46 (2)	C3—C4—H4B	109.5
Cl1^i^—Ni1—S1	112.96 (2)	H4A—C4—H4B	109.5
S1^i^—Ni1—S1	94.55 (3)	C3—C4—H4C	109.5
C1—S1—Ni1	99.91 (6)	H4A—C4—H4C	109.5
C1—N1—C3	109.97 (16)	H4B—C4—H4C	109.5
C1—N1—C2	124.69 (16)	C3—C5—N2	106.67 (17)
C3—N1—C2	125.30 (17)	C3—C5—C6	130.91 (18)
C1—N2—C5	109.99 (16)	N2—C5—C6	122.42 (18)
C1—N2—C7	125.03 (17)	C5—C6—H6A	109.5
C5—N2—C7	124.97 (17)	C5—C6—H6B	109.5
N2—C1—N1	106.54 (16)	H6A—C6—H6B	109.5
N2—C1—S1	127.15 (14)	C5—C6—H6C	109.5
N1—C1—S1	126.23 (15)	H6A—C6—H6C	109.5
N1—C2—H2A	109.5	H6B—C6—H6C	109.5
N1—C2—H2B	109.5	N2—C7—H7A	109.5
H2A—C2—H2B	109.5	N2—C7—H7B	109.5
N1—C2—H2C	109.5	H7A—C7—H7B	109.5
H2A—C2—H2C	109.5	N2—C7—H7C	109.5
H2B—C2—H2C	109.5	H7A—C7—H7C	109.5
C5—C3—N1	106.84 (16)	H7B—C7—H7C	109.5
			
Cl1^i^—Ni1—S1—C1	−63.57 (7)	C1—N1—C3—C5	0.2 (2)
Cl1—Ni1—S1—C1	66.74 (7)	C2—N1—C3—C5	−177.55 (18)
S1^i^—Ni1—S1—C1	−176.95 (7)	C1—N1—C3—C4	−179.83 (17)
C5—N2—C1—N1	−0.2 (2)	C2—N1—C3—C4	2.4 (3)
C7—N2—C1—N1	178.56 (17)	N1—C3—C5—N2	−0.3 (2)
C5—N2—C1—S1	−177.22 (14)	C4—C3—C5—N2	179.72 (19)
C7—N2—C1—S1	1.6 (3)	N1—C3—C5—C6	179.12 (19)
C3—N1—C1—N2	0.0 (2)	C4—C3—C5—C6	−0.9 (4)
C2—N1—C1—N2	177.77 (17)	C1—N2—C5—C3	0.3 (2)
C3—N1—C1—S1	177.06 (14)	C7—N2—C5—C3	−178.45 (18)
C2—N1—C1—S1	−5.2 (3)	C1—N2—C5—C6	−179.15 (17)
Ni1—S1—C1—N2	−89.72 (16)	C7—N2—C5—C6	2.1 (3)
Ni1—S1—C1—N1	93.84 (16)		

Symmetry code: (i) −*x*, *y*, −*z*+1/2.
