# Supplementary figures and images for: Crystal structure of di­chlorido­bis­(1,3,4,5-tetra­methyl-1H-imidazol-2-ium-2-thiol­ate-κS)nickel(II)
Source: Acta Crystallogr E Crystallogr Commun. 2015 Jul 4;71(Pt 8):m147. doi: 10.1107/S2056989015012281 (PMC4571377; doi:10.1107/S2056989015012281)

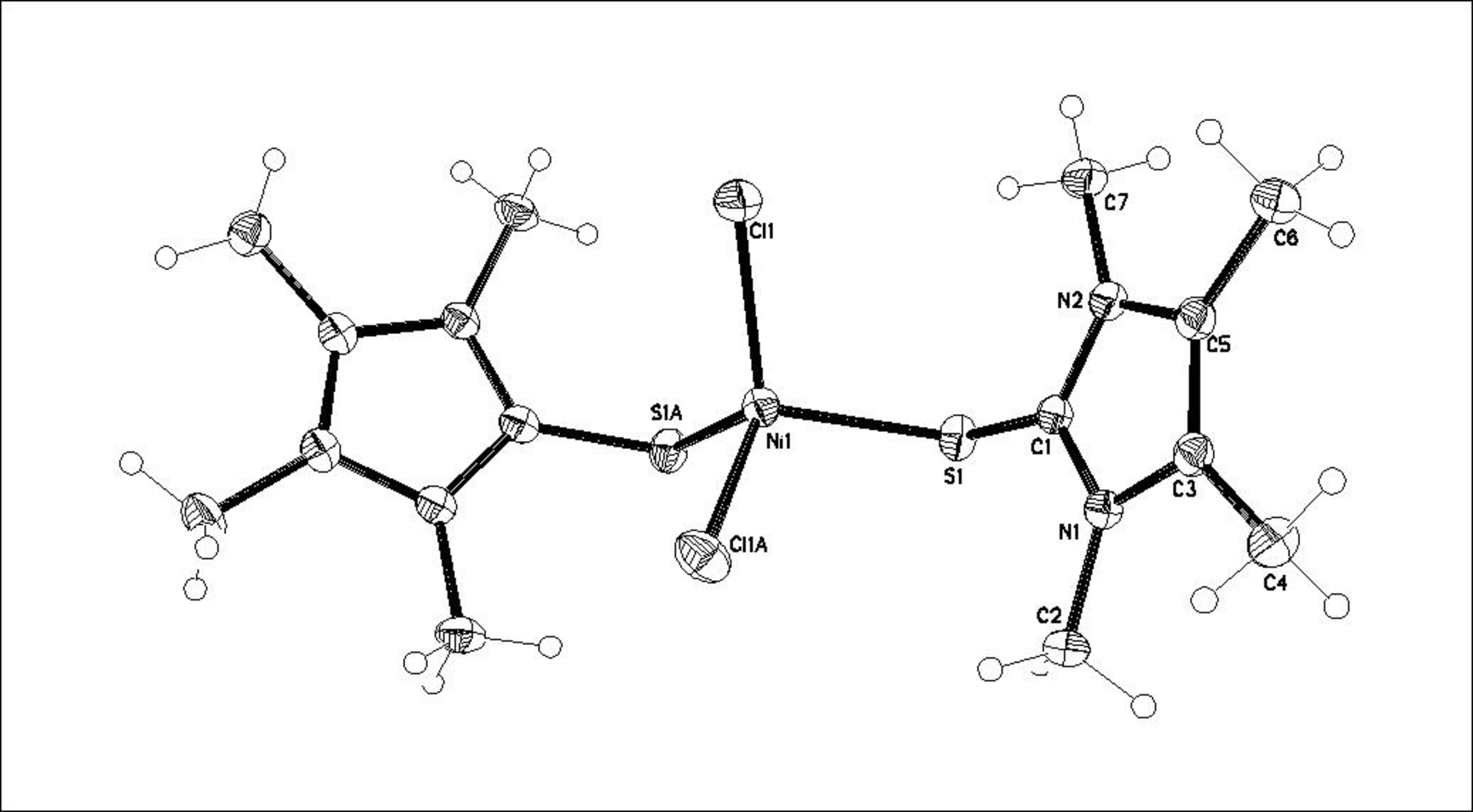

Supplement: Supplementary file 3 [file e-71-0m147-fig1.tif]
